# Genes Involved in miRNA Biogenesis Are Not Downregulated in SARS-CoV-2 Infection

**DOI:** 10.3390/v15051177

**Published:** 2023-05-16

**Authors:** Nathalie Garnier, Famara Sane, Layal Massara, Fabrice Soncin, Philippe Gosset, Didier Hober, Sabine Szunerits, Ilka Engelmann

**Affiliations:** 1 Laboratoire de Virologie ULR3610, University Lille and CHU Lille, F-59000 Lille, France; 2 Univ. Lille, CNRS, Centrale Lille, Univ. Polytechnique Hauts-de-France, UMR 8520, IEMN, F-59000 Lille, France; 3 CNRS UMR 9017, Inserm U1019, CHU Lille, Institut Pasteur de Lille, CIIL—OpInfIELD, University Lille, F-59000 Lille, France; 4 CNRS/IIS/Centre Oscar Lambret/Lille University SMMiL-E Project, CNRS Délégation Hauts-de-France, F-59000 Lille, France; 5 Laboratory for Integrated Micro Mechatronic Systems, Institute of Industrial Science, University of Tokyo, CNRS IRL2820, Tokyo 113-0033, Japan; 6 PCCEI, University Montpellier, INSERM, EFS, CHU Montpellier, F-34000 Montpellier, France

**Keywords:** miRNA, SARS-CoV-2, RNA interference, COVID-19, Ago2, Dicer, DGCR8, Drosha, Exportin-5

## Abstract

miRNAs, small non-coding RNAs that regulate gene expression, are involved in various pathological processes, including viral infections. Virus infections may interfere with the miRNA pathway through the inhibition of genes involved in miRNA biogenesis. A reduction in the number and the levels of miRNAs expressed in nasopharyngeal swabs of patients with severe COVID-19 was lately observed by us, pointing towards the potential of miRNAs as possible diagnostic or prognostic biomarkers for predicting outcomes among patients with severe acute respiratory syndrome coronavirus-2 (SARS-CoV-2) infection. The objective of the present study was to investigate whether SARS-CoV-2 infection influences the expression levels of messenger RNAs (mRNAs) of key genes involved in miRNA biogenesis. mRNA levels of *AGO2*, *DICER1*, *DGCR8*, *DROSHA*, and Exportin-5 (*XPO5*) were measured by quantitative reverse-transcription polymerase chain reaction (RT-qPCR) in nasopharyngeal swab specimens from patients with COVID-19 and controls, as well as in cells infected with SARS-CoV-2 in vitro. Our data showed that the mRNA expression levels of *AGO2*, *DICER1*, *DGCR8*, *DROSHA*, and *XPO5* were not significantly different in patients with severe COVID-19 when compared to patients with non-severe COVID-19 and controls. Similarly, the mRNA expression of these genes was not affected by SARS-CoV-2 infection in NHBE and Calu-3 cells. However, in Vero E6 cells, *AGO2*, *DICER1*, *DGCR8*, and *XPO5* mRNA levels were slightly upregulated 24 h after infection with SARS-CoV-2. In conclusion, we did not find evidence for downregulation of mRNA levels of miRNA biogenesis genes during SARS-CoV-2 infection, neither ex vivo nor in vitro.

## 1. Background

A novel coronavirus, named severe acute respiratory syndrome coronavirus-2 (SARS-CoV-2) emerged in 2019 [1]. It rapidly spread around the globe and caused a pandemic with more than 670 million cases and more than 6.8 million deaths as of 10 March 2023 [2]. The end of COVID-19 as a public health emergency of international concern was recently declared by the head of the World Health Organization (WHO), however, stating that this does not mean that the disease is no longer a global threat [3]. The pandemic has had profound impacts on society and economy due to containment measures, including travel restrictions, school closures, and even complete lock-downs [4]. It also has shed light on the importance of having access to rapid and accurate diagnostics; however, massive diagnostic testing led to shortages in several reagents and plasticware needed for performing RT-PCR for SARS-CoV-2 detection [5,6].

SARS-CoV-2, the etiological agent of coronavirus disease 2019 (COVID-19) [7], belongs to the genus *Betacoronavirus* of the *Coronaviridae* family [8] and is an enveloped virus with a positive-sense single-stranded RNA genome of approximately 30 kb [9]. Severe forms of this disease are characterized by a state of hyperinflammation called ‘cytokine storm’ and are associated with severe lung disease with acute respiratory distress syndrome (ARDS) [10,11]. Several symptoms can be present, such as fatigue, fever, cough, dyspnea, headache, conjunctivitis, sore throat, dysgeusia, hyposmia, and gastrointestinal symptoms such as diarrhea, nausea, and vomiting [12,13]. Despite the development of vaccines and the availability of some treatment options, the understanding of the pathophysiology of the infection still remains a priority, especially due to observed symptoms of post-acute sequelae of SARS-CoV-2 infection (PASC), also known as “long COVID”, possibly related to a persistence of SARS-CoV-2 and reactivation of the latent pathogen [14]. Regarding the pathophysiology, several studies have shown that miRNAs are involved in infections caused by other coronaviruses [15,16,17,18,19,20]. microRNAs have also been shown to be involved in the pathophysiology of SARS-CoV-2 infection [21,22,23,24].

RNA silencing is a fundamental cellular mechanism of gene regulation and includes the small interfering RNA (siRNA) and microRNA (miRNA) pathways [25,26]. The miRNA pathway is evolutionarily conserved in metazoans [27]. miRNAs are small non-coding RNAs of approximately 22 nucleotides that regulate gene expression at the post-transcriptional level. miRNAs exert their function by binding most commonly to the 3′ untranslated regions (UTRs) or alternatively to 5′ UTRs or in the open reading frames (ORFs) of their target mRNAs [27,28,29]. miRNA biogenesis begins with the transcription from genes encoding the miRNA in the nucleus, resulting in a primary transcript with a hairpin structure, named pri-miRNA [30]. Then, the loop end of the pri-miRNA is cleaved by the microprocessor complex formed by the ribonuclease III Drosha and the co-factor DGCR8 (DiGeorge syndrome critical region gene 8) [31]. The cleavage results in the generation of the precursor miRNA, named pre-miRNA, which has a hairpin structure of about 70 nucleotides [32]. Next, the pre-miRNA is exported into the cytoplasm through Exportin-5 [31]. In the cytoplasm, the pre-miRNA is cleaved by Dicer, a cytoplasmic RNase endonuclease, to form the mature miRNA duplex of around 22 nucleotides [33]. This mature duplex miRNA associates with one argonaute protein (AGO), and following the expulsion of one of the miRNA strands, termed the passenger strand, the remaining single-stranded guide miRNA becomes part of the miRNA-induced silencing complex (miRISC) [34]. This complex allows the post-transcriptional regulation of gene expression through two mechanisms: translational repression or mRNA degradation [34,35,36].

The total number of human miRNAs is estimated to be 2300 [37]. miRNAs regulate the expression of most human genes and are involved in several physiological and pathological processes, such as development, cancers, viral infections, and antiviral immune responses [27]. Viruses interact with the miRNA machinery of their hosts and this interaction can either result in an increase or a repression of the expression of specific miRNAs. Thus virus infections profoundly affect cellular miRNA expression profiles [38,39,40,41,42]. Furthermore, several viruses encode their own viral miRNAs [38,39,41]. On the other hand, viruses can also globally interfere with the miRNA pathway. In fact, in plants and insects, RNA silencing pathways function as antiviral defense mechanisms [43,44]. Recent findings suggest they may also have antiviral functions in mammalian cells [44,45]. To escape this antiviral defense, plant and insect viruses possess virus-encoded suppressors of RNA interference that modulate the activity of central components of the RNA silencing pathways in order to favor viral replication or to inhibit host antiviral defense mechanisms [39,44,46]. There is some evidence that viruses also interfere with the miRNA pathway in mammals [39,44,47,48].

Several studies have shown a difference in miRNA expression levels between COVID-19 patients and uninfected individuals [49,50,51,52,53,54,55,56,57,58,59,60,61,62]. In our previous study [55], a lower total number of miRNAs expressed in nasopharyngeal swabs of patients with severe COVID-19 was noted. Furthermore, most differentially regulated miRNAs were downregulated in severe COVID-19 patients [55]. Among the hypotheses put forward to explain this observation was that SARS-CoV-2 infection inhibited cellular miRNA biogenesis. In the current study, we therefore investigated the impact of SARS-CoV-2 infection on the expression of miRNA biogenesis genes.

## 2. Materials and Methods

### 2.1. Patients and Specimens

Nasopharyngeal swab specimens of 60 patients were used in the study, including 19 patients with severe COVID-19, 21 patients with non-severe COVID-19 and 20 patients without COVID-19 (control group) [55]. Patients with severe COVID-19 needed intensive care unit admission and oxygen treatment and patients with non-severe COVID-19 required neither intensive care nor oxygen treatment. Nasopharyngeal swab specimens were obtained for routine diagnostic purposes by using flocked swabs that were placed in universal transport medium. Nasopharyngeal swab specimens were stored at −80°C. The study was approved by the French Institutional Authority for Personal Data Protection (Commission Nationale de l’Informatique et des Libertés DR-2020-178, 22 October 2020) and the ethics committee (Comité de Protection des Personnes Nord Ouest IV, ECH20/09, 7 September 2020).

### 2.2. Cells

The Vero E6 cell line (ATCC, CRL-1586) is a clone of strain 76, isolated from the kidney of an African green monkey with the morphology of epithelial cells. The Calu-3 cell line (Merck #SCC438, Sigma Aldrich, Saint-Quentin-Fallavier, France) is an epithelial cell line derived from human lung adenocarcinoma. The Vero E6 and Calu-3 cell lines were cultivated with DMEM medium, 4.5 g/L glucose, 10% fetal calf serum, 1% L-glutamine, and 1% penicillin and streptomycin (Gibco #15070-063, Thermofisher Scientific, Courtaboeuf, France) at 37 °C in 95% air/5% CO_2_ atmosphere.

Normal Human Bronchial Epithelial (NHBE) (Lonza, Switzerland) are human epithelial cells isolated from the airway epithelium above the bifurcation of the lungs of healthy patients. They were cultured with PneumaCultTM-Ex Plus Medium (StemCell Technologies, Saint Égrève, France) at 37 °C in 95% air/5% CO_2_ atmosphere.

### 2.3. Virus

SARS-CoV-2, variant B.1.617.2 (delta variant, GISAID ID EPI_ISL_2143633) was isolated from a clinical specimen and cultivated on Vero E6 cells in a biosafety 3 (BSL3) facility.

### 2.4. Infection of Cells

For Vero E6 cells, 10^4^ cells per well were plated in 96-well plates two days before infection. Four replicates were used. For Calu-3 cells, 10^5^ cells per well were plated in 48-well plates one day before infection. Four replicates were used. For NHBE cells, 5 × 10^5^ cells per well were plated in 6-well plates one day before infection. Four replicates were used. Cells were infected with SARS-CoV-2 with a multiplicity of infection (MOI) of 0.1 for 24 and 48 h, with incubation of cells with the virus for 1 h at 37 °C in 95% air/5% CO_2_ atmosphere, followed by three washes with DMEM culture medium.

### 2.5. RNA Extraction

RNA was extracted from nasopharyngeal swab specimens using the MagMAX mirVana Total RNA Isolation Kit (Thermofisher Scientific, Courtaboeuf, France) according to the manufacturer’s instructions.

RNA extraction from NHBE and Vero E6 cells was performed using miRNAeasy Tissue/Cells Advanced Micro Kit (QIAGEN, Courtaboeuf, France) according to the manufacturer’s instructions. RNA extraction from Calu-3 cells was performed using a QIAamp Viral RNA kit (QIAGEN, Courtaboeuf, France) according to the manufacturer’s instructions. RNA extracts were treated with DNAse (Jena Bioscience, Germany) for 10 min at 37 °C followed by 10 min at 65 °C. RNA extracts were stored at −80 °C.

### 2.6. RT-qPCR

The primer sequences for *AGO2* (PrimerBank ID: 257467481c1), *DICER1* (PrimerBank ID: 307133774c1), *DGCR8* (PrimerBank ID: 298358603c1), *DROSHA* (PrimerBank ID: 155030235c1), and *XPO5* (PrimerBank ID: 221136812c1) genes (Table 1) were retrieved from the PrimerBank of Harvard Medical School [63]. RT-qPCR was performed to quantify mRNA expression of the genes involved in miRNA biogenesis by using 5 ng of extracted RNA of cells and 5 microL of RNA extracts of patients’ specimens, respectively, and the Sybr green Luna Universal one-step RT-qPCR kit (New England BioLabs, Evry, France) according to the manufacturer’s instructions using a 7500 Real-Time PCR System (ThermoFisher Scientific, Courtaboeuf, France). The following thermal profile was used: 10 min at 55 °C followed by 1 min at 95 °C and 40 cycles of 10 s at 95 °C, 30 s at 53 °C and 60 s at 60 °C. The beta-actin (*ACTB* gene) was used for normalization. Results were analyzed by using the 7500 Software (v2.0.6, Life Technologies, Thermofisher Scientific, Courtaboeuf, France). Results were presented as delta Ct values = Ct of the gene of interest − Ct of beta-actin. Fold changes were calculated according to the 2 ^−delta delta Ct^ method, with delta delta Ct = delta Ct (infected cells)—delta Ct (uninfected cells) [64].

### 2.7. Statistical Analysis

Statistical analysis was performed with Prism 9 for Windows (Version 9.5.1) using nonparametric and unpaired tests; Kruskal–Wallis test and Mann–Whitney U test. The results were considered significant when the *p*-value was below 0.05.

## 3. Results

### 3.1. Expression of miRNA Biogenesis Genes in COVID-19 Patients’ Specimens

**Figure 1:**
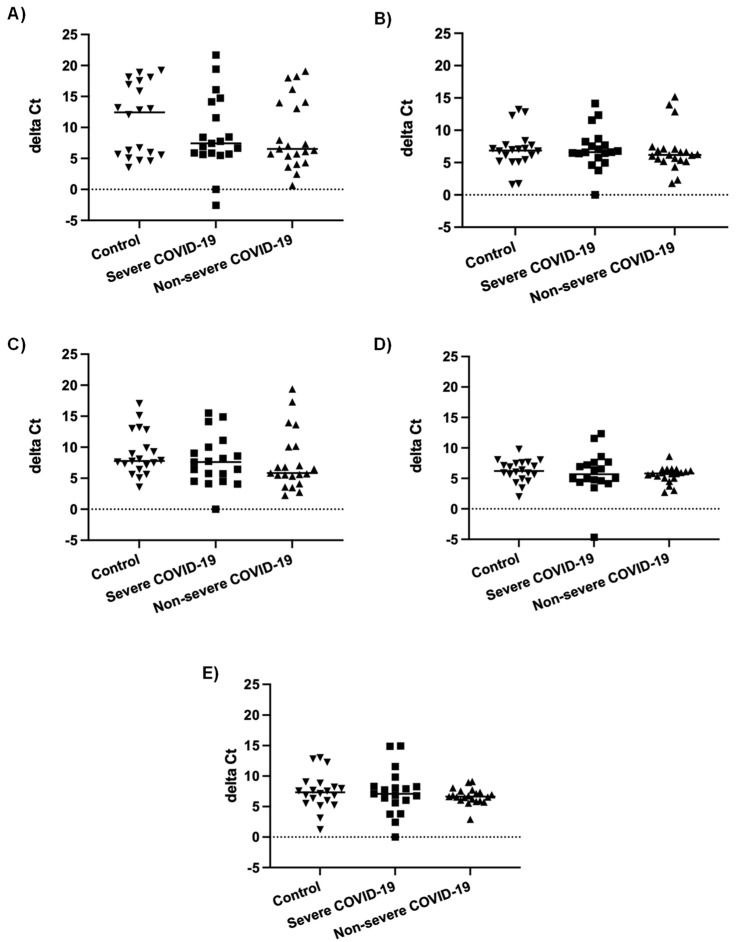
mRNA expression levels of genes involved in miRNA biogenesis. Normalized mRNA expression (delta Ct values) of *AGO2* (**A**), *DICER1* (**B**), *DGCR8* (**C**), *DROSHA* (**D**), and *XPO5* (**E**) genes in nasopharyngeal swabs of patients with severe COVID-19 (n = 19), patients with non-severe COVID-19 (n = 21) and controls (n = 20). Each data point represents one nasopharyngeal swab specimen. The bar indicates the median value.

In our previous study, the number of miRNAs detected in nasopharyngeal swabs of severe COVID-19 patients was lower than in non-severe COVID-19 patients and controls. Furthermore, most differentially expressed miRNAs were downregulated in severe COVID-19 patients compared to patients with non-severe COVID-19 and controls [55]. In order to determine whether this could be due to an inhibition of miRNA biogenesis during severe COVID-19, we compared mRNA expression of genes involved in miRNA biogenesis, namely *AGO2*, *DICER1*, *DGCR8*, *DROSHA*, and *XPO5*, in nasopharyngeal swabs of severe and non-severe COVID-19 patients and controls. As shown in Figure 1, there were no statistically significant differences in mRNA levels of these genes between the three different patient groups.

### 3.2. Expression of miRNA Biogenesis Genes in SARS-CoV-2 Infected Cells

**Figure 2:**
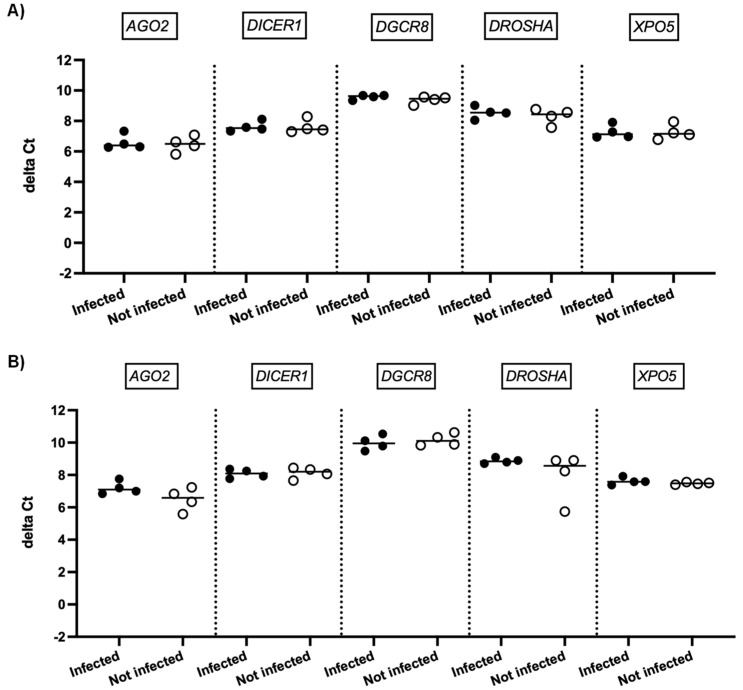
mRNA expression of genes involved in miRNA biogenesis in NHBE cells. Normalized mRNA levels (delta Ct values) of *AGO2*, *DICER1*, *DGCR8*, *DROSHA*, and *XPO5* genes were measured in NHBE cells infected with SARS-CoV-2 (black circles) or in non-infected cells (empty circles), 24 h (**A**) and 48 h (**B**) post infection. Four independent infections are shown, each data point represents one replicate. The bar indicates the median.

**Figure 3:**
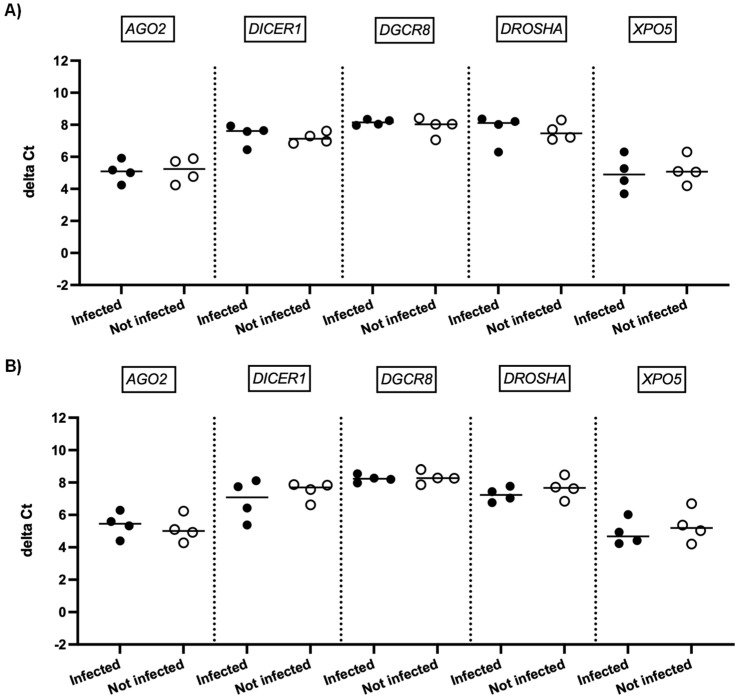
mRNA expression of genes involved in miRNA biogenesis in Calu-3 cells. Normalized mRNA levels (delta Ct values) of *AGO2*, *DICER1*, *DGCR8*, *DROSHA*, and *XPO5* genes were measured in Calu-3 cells infected with SARS-CoV-2 (black circles) or in non-infected cells (empty circles) 24 h (**A**) and 48 h (**B**) post infection. Four independent infections are shown. Each data point represents one replicate. The bar indicates the median.

**Figure 4:**
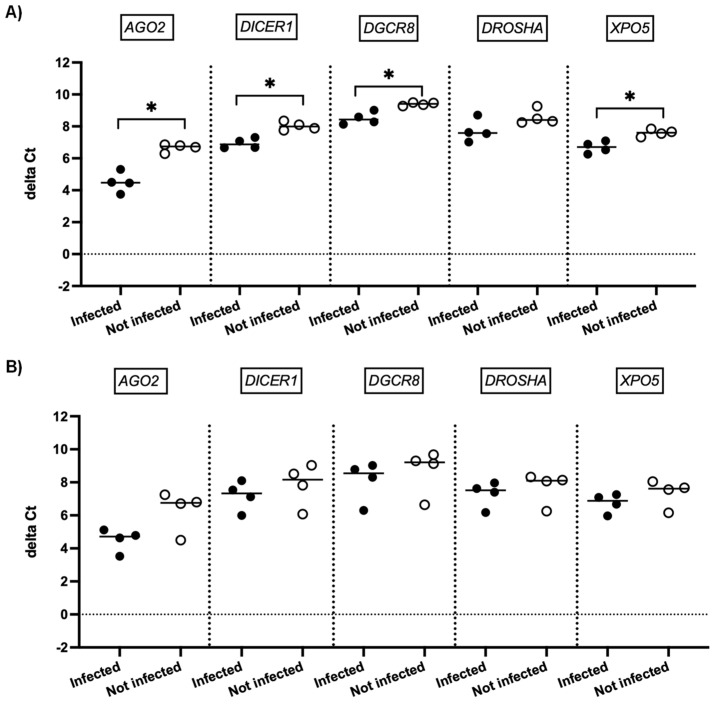
mRNA expression of genes involved in miRNA biogenesis in Vero E6 cells. Normalized mRNA levels (delta Ct values) of *AGO2*, *DICER1*, *DGCR8*, *DROSHA,* and *XPO5* genes were measured in Vero E6 cells infected with SARS-CoV-2 (black circles) or in non-infected cells (empty circles), 24 h (**A**) and 48 h (**B**) post infection. Four independent infections are shown. Each data point represents one replicate. The bar indicates the median. * *p* < 0.05.

**Figure 5:**
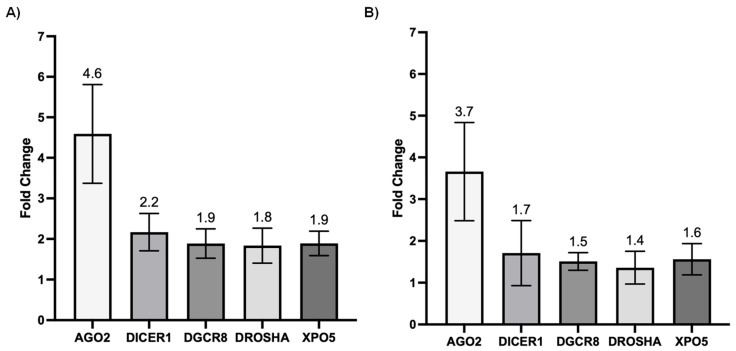
Fold changes of mRNA expression of genes involved in miRNA biogenesis in infected Vero E6 cells. Fold changes of mRNA expression of *AGO2*, *DICER1*, *DGCR8*, *DROSHA*, and *XPO5* genes in SARS-CoV-2 infected compared to uninfected Vero E6 cells at 24 h (**A**) and 48 h (**B**) post infection. Means and standard deviations of four independent infections are shown.

We next investigated whether SARS-CoV-2 infection impacted the expression of genes involved in miRNA biogenesis in vitro. To this end, primary cultures of human bronchial epithelial (NHBE) cells were used as they represent a cellular model close to the human respiratory tract. In addition, two epithelial cell lines that are commonly used for in vitro studies concerning SARS-CoV-2, namely Calu-3 and Vero E6 cell lines, were also included in this study. The three cell types were infected with the SARS-CoV-2 delta variant at an MOI of 0.1, and mRNA expression levels of *AGO2*, *DICER1*, *DGCR8*, *DROSHA*, and *XPO5* were measured 24 h and 48 h after infection. In parallel, we confirmed that SARS-CoV2 effectively infected these cells by measuring the viral RNA production (data not shown). Again, as shown in Figure 2 and Figure 3, no significant differences in mRNA levels of these genes were observed after infection of NHBE and Calu-3 cells with SARS-CoV-2 at 24 h and 48 h post infection. However, as shown in Figure 4, mRNA levels of miRNA biogenesis genes were impacted by SARS-CoV-2 infection in Vero E6 cells at 24 h post infection. A significant increase in mRNA levels of *AGO2*, *DICER1*, *DGCR8*, and *XPO* (all *p*-values= 0.03) was found 24 h after infection (Figure 4A). In contrast, 48 h after infection, no significant differences in mRNA levels of these genes were observed (Figure 4B). We next calculated the fold change expression of mRNA levels in infected Vero E6 cells. Indeed, mRNA levels were higher in infected Vero E6 cells as compared to uninfected cells (Figure 5). mRNA level changes of *DICER1*, *DGCR8*, and *XPO* were approximately 2-fold, and of *AGO2* approximately 4.6-fold, 24 h after infection. Thus, we observed a slight and transient overexpression of mRNA levels of key miRNA biogenesis genes after SARS-CoV-2 infection in Vero E6 cells at 24 h post infection but not at 48 h post infection.

## 4. Discussion

While a reduced number of miRNAs was expressed in nasopharyngeal swabs specimens of patients with severe COVID-19 compared to non-severe COVID-19 patients and controls, and most miRNAs were downregulated in severe COVID-19 patients [55], the reasons for these observations were not clear. The inhibition of miRNA biogenesis during SARS-CoV-2 infection was considered to be the most likely underlying reason, as supported by a study by Mousavi et al., who noted that *AGO2*, *DICER*, and *DROSHA* were downregulated in COVID-19 patients compared to controls [65] and suggested that viruses may interact with the miRNA biogenesis pathway [44,46].

A dysregulation of the expression of genes involved in miRNA biogenesis had actually been found in several other viral infections: Dengue virus infection led to a decrease of mRNA levels of *DICER*, *DROSHA*, *AGO1*, and *AGO2* in Huh-7 cells and this was associated with increased viral replication [66]. In another study, infection of A549 cells with dengue virus 4 resulted in reduced mRNA levels of *DICER*, *DROSHA*, and *DGCR8* [67]. Vaccinia virus infection led to a general decrease of miRNA expression in infected cells and was associated with a decrease of *DICER* expression at the mRNA and protein levels [68]. Influenza virus A infection also led to a decrease of *DICER* mRNA and protein levels in infected A549 and Vero cells [69]. Interestingly, *DROSHA* mRNA levels were not impacted by vaccinia virus infection. In contrast, infection with herpes simplex type 1 and type 2, influenza A virus, and human respiratory syncytial virus had no effect on *DICER* expression [68], suggesting that the impact of virus infection on the expression of genes involved in miRNA biogenesis differs between viruses. One study even observed a different impact of yellow fever virus genotype I versus genotype II on mRNA levels of miRNA biogenesis components in infected cells [70].

Furthermore, most studies investigated the effect of virus infection on miRNA biogenesis genes by using in vitro infected cells. To date, only a few studies have investigated this effect in clinical samples. Apart from the above-mentioned study [65], one study investigated miRNA expression and expression of miRNA biogenesis genes in HTLV-1 infected patients [71]. Interestingly, the authors measured *DROSHA*, *DGCR8*, *XPO5*, *DICER1*, *AGO2*, and *AGO3* mRNA expression, and only *DICER1* mRNA was differently expressed in CD8^+^ T-cell–depleted PBMCs from HTLV-1 asymptomatic carriers when compared to patients with acute adult T cell Leukemia. This led to a reduction in the expression level of several miRNAs [71]. Another study found that patients with chronic hepatitis B who had high hepatitis B virus loads had reduced mRNA levels of *DROSHA*, *DICER1*, and *AGO2* compared with patients with low virus loads [72]. Interestingly, reduced mRNA expression levels of *DICER*, *DROSHA*, and *AGO2* were also observed in hepatitis B virus replicon-transfected HepG2 cells [72].

In the present study, we investigated the expression levels of genes implicated in miRNA biogenesis both in vitro and ex vivo. Measured mRNA levels of *AGO2*, *DICER1*, *DGCR8*, *DROSHA*, and *XPO5* were not significantly different in nasopharyngeal swab specimens of severe COVID-19 patients compared to non-severe COVID-19 patients or controls (Figure 1). No impact of SARS-CoV-2 infection on mRNA expression levels of key genes involved in miRNA biogenesis was observed, in contrast to the study by Mousavi et al., who found that mRNA levels of *AGO2*, *DICER1,* and *DROSHA,* but not *DGCR8*, differed between COVID-19 patients and controls [65]. There are several differences between our study and the one from Mousavi et al. First, in the present study, we used nasopharyngeal swab specimens, whereas Mousavi et al. used whole blood specimens. Second, there were differences in the experimental protocols: Mousavi et al. used RT followed by qPCR, whereas we used one-step RT qPCR. The primer sequences used in the two studies were not the same. The gene used for normalization was GAPDH in the study by Mousavi et al., whereas we used beta-actin. While all of these differences may have an impact on the results, the most likely explanation for the discrepancy in the findings of the two studies is, in our opinion, the fact that we measured mRNA expression in very different specimen types [65]. When investigating mRNA expression in nasopharyngeal swabs, we studied the local effect of SARS-CoV-2 infection directly on the targeted respiratory epithelium and confirmed that in vitro SARS-CoV2 infection of airway epithelial cells did not affect mRNA expression of these genes. In contrast, measuring mRNA expression in the blood reflected a systemic effect of infection in leucocytes, which may or may not have been caused by SARS-CoV-2 infection directly. Of note, it was not mentioned whether SARS-CoV-2 viruses had been detected in the patients’ blood [65].

The impact of SARS-CoV-2 infection on the expression of genes involved in miRNA biogenesis in in vitro models was tested to validate the clinical observations. The results showed that infection of NHBE and Calu-3 cells with SARS-CoV-2 did not impact mRNA expression levels of *AGO2*, *DICER1*, *DGCR8*, *DROSHA*, and *XPO5* at 24 and 48 h post infection (Figure 2 and Figure 3), in good concordance with our results on patient samples. On the other hand, infection of Vero E6 cells with SARS-CoV-2 impacted mRNA levels of *AGO2*, *DICER1*, *DGCR8*, and *XPO5* at 24 h (Figure 4A). However, rather than being downregulated as we had hypothesized, mRNA levels were slightly and transiently increased in SARS-CoV-2 infected cells at 24 h post infection. mRNA expression changes were around two-fold in most cases (Figure 5), and even if differences were statistically significant, it is uncertain whether these slight changes have a biological impact. Furthermore, at 48 h post infection, mRNA levels were not significantly different between SARS-CoV-2 infected and uninfected cells (Figure 4B). Indeed, increased mRNA expression of genes involved in miRNA biogenesis has been observed in other virus infections. For example, increased *DICER1* and *DROSHA* mRNA expression were found in some human papillomavirus (HPV) positive cervical cancer cell lines [73]. Expression of the HPV E6 and E7 oncoproteins in primary human foreskin keratinocytes resulted in the upregulation of DICER1 mRNA and DROSHA mRNA expression [73], suggesting that these viral proteins are responsible for the induction of gene expression. Another example is that infection with Kaposi’s sarcoma-associated herpesvirus (KSHV) resulted in increased expression of *DICER1* mRNA in primary human umbilical vein endothelial cells, whereas mRNA expression levels of *DGCR8*, *DROSHA*, and *XPO5* did not change significantly [74]. The authors hypothesized that KSHV induced upregulation of *DICER1* expression in order to counteract the miRNA biogenesis inhibition caused by the human MCP-1-induced protein-1 [74].

Taken together, we found no evidence that SARS-CoV-2 infection inhibited miRNA biogenesis by downregulation of mRNA levels of key miRNA biogenesis genes. However, the fact that the mRNA levels of genes involved in miRNA biogenesis were not downregulated after SARS-CoV-2 infection does not necessarily mean that the miRNA pathway is not affected by SARS-CoV-2 infection. There are several alternative mechanisms by which SARS-CoV-2 infection may interfere with the miRNA pathway [39]. For example, there could be a direct interaction of viral factors with central components of the miRNA pathway, leading to their inhibition. Indeed it was shown that human adenovirus virus-associated RNAs inhibited DICER activity [47]. Similarly, the insect flock house virus B2 protein interacted with DICER and thereby inhibited siRNA biogenesis [75]. Furthermore, the Zika virus capsid interacted with DICER and inhibited miRNA biogenesis [76]. Interestingly, and in parallel to our observation of reduced miRNA expression in severe COVID-19 [55], Zika virus infection of neural stem cells resulted in a reduction of total miRNA reads, and 138 miRNAs were significantly downregulated; in contrast, only two miRNAs were upregulated [76]. HSV-1 used a different mechanism to interfere with miRNA biogenesis, namely by blocking pre-miRNA nuclear export [77]. Reduced expression of mature miRNAs can also be explained by cleavage of miRNA precursors. Indeed, human MCP-1-induced protein-1 cleaved the terminal loops of pre-miRNAs leading to the destabilization of pre-miRNAs and resulting in their degradation [74,78]. Increased turnover of mature miRNAs could also underlie a reduced miRNA expression [39,74,79]. For example, poxvirus-encoded poly(A)-polymerase mediated poly-adenylation of cellular miRNAs, resulting in their degradation. This phenomenon was observed in insect and mammalian cells. Interestingly, restoring miRNA function resulted in reduced virus replication suggesting that the virus-induced degradation of host miRNAs favored virus replication [80].

Altogether, these findings show that many viruses interact with the miRNA pathway and that the mechanisms used are different. Concerning SARS-CoV-2 infection, the possible mechanisms need to be explored in future studies.

## 5. Conclusions

Taken together, our results suggest that there is no detectable downregulation of mRNA expression of genes involved in miRNA biogenesis during SARS-CoV-2 infection, neither ex vivo nor in vitro.

## Figures and Tables

**Table 1:** List and sequences of primers.

Name of Oligonucleotides	Sequence (5′-3′)	
*AGO2*	Forward	TCCACCTAGACCCGACTTTGG
	Reverse	GTGTTCCACGATTTCCCTGTT
*DICER1*	Forward	GAGCTGTCCTATCAGATCAGGG
	Reverse	ACTTGTTGAGCAACCTGGTTT
*DGCR8*	Forward	GCAGAGGTAATGGACGTTGG
	Reverse	AGAGAAGCTCCGTAGAAGTTGAA
*DROSHA*	Forward	TGTCACAGAATGTCGTTCCAC
	Reverse	GGGCCTAAAGGATGGTGCT
*XPO5*	Forward	ATCCTGGAACACGTTGTCAAG
	Reverse	CACTACAATTCGAGACAGAGCAT
*ACTB*	Forward	TTGCCGACAGGATGCAGA
	Reverse	GCCGATCCACACGGAGTACT

## Data Availability

The data presented in this study are available on request from the corresponding author.
